# Intravesical gas explosion after sequential greenlight laser prostate vaporization and plasma electrocoagulation: a case report and literature review

**DOI:** 10.3389/fruro.2026.1845524

**Published:** 2026-09-09

**Authors:** Jia Wei, Qinya Zeng, Yangcai Wang, Guangming Yan

**Affiliations:** 1 Department of Anesthesiology, The Second Affiliated Hospital of Army Medical University, Chongqing, China; 2 Department of Urology, The Second Affiliated Hospital of Army Medical University, Chongqing, China

**Keywords:** benign prostatic hyperplasia, bladder rupture, case reports, GreenLight laser vaporization, intravesical gas explosion, plasma electrocoagulation

## Abstract

**Background:**

Intravesical gas explosion resulting in full-thickness intraperitoneal bladder rupture is an extremely rare but potentially life-threatening complication following transurethral prostate surgery, with very few cases reported in the context of Greenlight laser vaporization of the prostate (LVP) and plasma electrocoagulation.

**Case presentation:**

A 50-year-old man with chronic urinary retention and benign prostatic hyperplasia (BPH) underwent LVP. During hemostasis, a sudden loud popping sound was noted, followed by an intravesical explosion and subsequent bladder rupture. Emergent laparoscopic exploration confirmed a full-thickness intraperitoneal bladder rupture, which was surgically repaired. The patient required an 11-day intensive care unit stay and prolonged urethral catheterization due to reduced bladder capacity. At 1-year follow-up, the catheter had been successfully removed, and the patient remained under conservative follow-up.

**Conclusion:**

Intravesical gas explosion is a rare but potentially severe complication of transurethral prostate surgery. This case demonstrates that the complication may occur during hemostasis following LVP and highlights the importance of intraoperative vigilance and preventive measures.

## Introduction

BPH is one of the most common urological conditions. Although many minimally invasive alternatives exist, transurethral resection of the prostate (TURP) remains the gold standard for surgical management of BPH (1). Common complications associated with TURP include bleeding, capsular perforation, mucosal injury, and transurethral resection syndrome (2).

LVP is a well-established alternative to TURP, offering effective tissue ablation with a favorable safety profile (3). The procedure utilizes a 532-nm potassium-titanyl-phosphate (KTP) or lithium triborate (LBO) laser enabling efficient vaporization with excellent hemostatic properties (4). Compared with TURP, LVP is associated with shorter catheterization and hospitalization times, as well as lower rates of bleeding-related complications and sexual dysfunction (5). Additionally, it has been shown to be safe and effective even in patients receiving anticoagulant therapy and those with large prostate volumes exceeding 80 g (6). However, recognized adverse effects include transient dysuria, hematuria, delayed bleeding, and a higher reoperation rate due to incomplete vaporization or regrowth of prostatic adenoma in the long term (7).

Intravesical gas explosion is an extremely rare but potentially life-threatening complication during TURP (8). The underlying mechanism involves ignition of explosive gas mixtures, primarily hydrogen, methane, and carbon monoxide, generated during tissue pyrolysis upon contact with the hot loop of a resectoscope (9). The introduction of oxygen into the bladder creates an explosive mixture with these flammable gases (10). Resulting injuries can range from superficial mucosal tears to full-thickness bladder rupture, the latter often requiring prompt surgical intervention (11).

Reported cases of intravesical gas explosion have predominantly been associated with TURP, with an incidence rate reported to range from 0.01% to 0.02% (8). Although such complications are uncommon in LVP, we report a rare case of intravesical gas explosion following LVP and plasma electrocoagulation. Based on the literature, we discuss the potential mechanisms, preventive strategies, and clinical implications to increase awareness and improve the prevention and management of this rare complication.

## Case report

### Patient characteristics

A 50-year-old man presented with a 3-month history of progressive urinary retention, hesitancy, weak stream, and nocturia, without gross hematuria or significant lower abdominal pain. His medical history included hypertension and dilated cardiomyopathy, both of which were well controlled with long-term medical therapy.

On physical examination, the patient had a blood pressure of 145/98 mmHg and a regular heart rate of 105 bpm. The IPSS was 25, consistent with severe lower urinary tract symptoms.

Laboratory findings were notable for a serum prostate-specific antigen (PSA) level of 4.33 ng/mL and an elevated serum creatinine level of 237.1 µmol/L (normal range: 45–105 µmol/L).

Abdominal ultrasonography demonstrated benign prostatic hyperplasia with an estimated prostate volume of 125 g and an overdistended bladder with considerable post-void residual urine; the bladder wall was intact, and no bladder diverticula, trabeculation, intravesical stones, or upper urinary tract dilatation were detected.

### Operative course

Following urethral catheterization for acute urinary retention, LVP was planned. The patient was placed in the lithotomy position. LVP was performed utilizing a third-generation Greenlight laser system (532 nm wavelength; J1 fiber, model 0010 2400; Boston Scientific Corporation, Marlborough, MA, USA) at a vaporization power of 160 W. Continuous irrigation with 0.9% normal saline was maintained throughout the procedure using a 26 F continuous-flow resectoscope sheath.

Prior to vaporization, cystoscopy revealed a markedly enlarged prostate with trilobar hyperplasia, patent ureteral orifices, and grossly normal bladder mucosa with mild trabeculation but no diverticula or stones. Tissue ablation was initiated at the elevated bladder neck and proceeded systematically through the median lobe and bilateral lateral lobes, with vaporization carried down to the prostatic capsule. During vaporization, significant bleeding from the prostatic tissue was encountered.

Due to poor visualization from persistent bleeding, plasma electrocoagulation was performed using a loop-electrode-equipped SIMAI plasma resectoscope system (SIMAI Medical Equipment, Zhuhai, China), with coagulation power of 60 W and cutting power of 80 W. Small prostatic tissue fragments were harvested for intraoperative frozen-section examination, followed by hemostasis. After evacuation of prostatic tissue fragments using an Ellik evacuator, scattered small air bubbles were observed at the 12 o’clock position of the prostatic fossa near the bladder neck. Intraoperative frozen-section analysis of the resected tissue raised concern for malignancy, and further histopathological examination was recommended.

### Explosion event and diagnosis

Approximately 15 minutes after initiation of the hemostasis phase, a sudden loud popping sound was heard from the lower abdomen, followed by ejection of irrigation fluid through the urethra. The patient immediately reported discomfort and dyspnea, with heart rate increasing to 126 bpm and blood pressure rising to 159/102 mmHg. The endoscope was reinserted for emergency cystoscopic examination. However, the irrigating fluid rapidly became blood-tinged and progressively more turbid, severely compromising visualization. Only a brief view was possible, revealing extensive mucosal disruption and a large defect on the anterior bladder wall, through which what appeared to be peritoneal fat was transiently visible, raising the suspicion of full-thickness bladder rupture.

Progressive lower-abdominal distension with percussion dullness ensued. After resectoscope and sheath removal, a 22 F three-way Foley catheter was placed for compression, yet hematuria persisted. Bedside ultrasound showed massive intra-abdominal free fluid, with pale-red fluid obtained on fine-needle paracentesis.

Intraperitoneal bladder rupture was suspected based on the sudden intraoperative event, abdominal distension, and intraperitoneal fluid accumulation. The temporal relationship between the intravesical explosion and bladder rupture supported the diagnosis of explosion-associated bladder injury. The total operative time before the explosion was approximately 100 minutes, including approximately 15 minutes of plasma electrocoagulation.

### Surgical repair and postoperative course

Laparoscopic exploration was performed via a three-port transperitoneal approach. A 10-mm supraumbilical port was used for the laparoscope, with two 5-mm working ports placed lateral to the rectus-sheath margin at the midpoint between the umbilicus and anterior superior iliac spine. Systematic abdominal survey of the liver, spleen, gastrointestinal tract, omentum and major retroperitoneal vessels revealed no intra-abdominal injuries other than bladder injury.

After evacuation of approximately 800 mL of blood-tinged fluid, the bladder was carefully inspected and mobilized. Three full-thickness lacerations were noted: (1) a 4.0-cm irregular, stellate-shaped laceration on the anterior wall near the bladder dome, with ragged hemorrhagic margins extending toward the dome; (2) an approximately 3.0-cm oblique linear laceration of the right lateral wall with relatively smooth margins; (3) an approximately 2.5-cm irregular laceration on the posterior wall superior to the trigone, with moderately ragged margins. There was no active bleeding from laceration edges, and no devitalized or nonviable tissue requiring debridement. The ureteral orifices and prostate capsule were confirmed intact.

The bladder lacerations underwent two-layer closure with 2-0 polyglactin: continuous mucosal approximation followed by interrupted seromuscular reinforcement. A 22-F transurethral three-way Foley catheter and suprapubic cystostomy tube were inserted. A pelvic drain was placed via the right lower port, and the abdomen was closed in layers.

### Follow-up

Postoperatively, bladder irrigation fluid gradually cleared. The Foley catheter was removed on day 7, the suprapubic cystostomy tube on day 10, and the pelvic drain on day 3. Because of persistent dysuria and reduced bladder capacity, an indwelling catheter was subsequently reinserted and maintained with regular exchanges.

**Figure 1:**
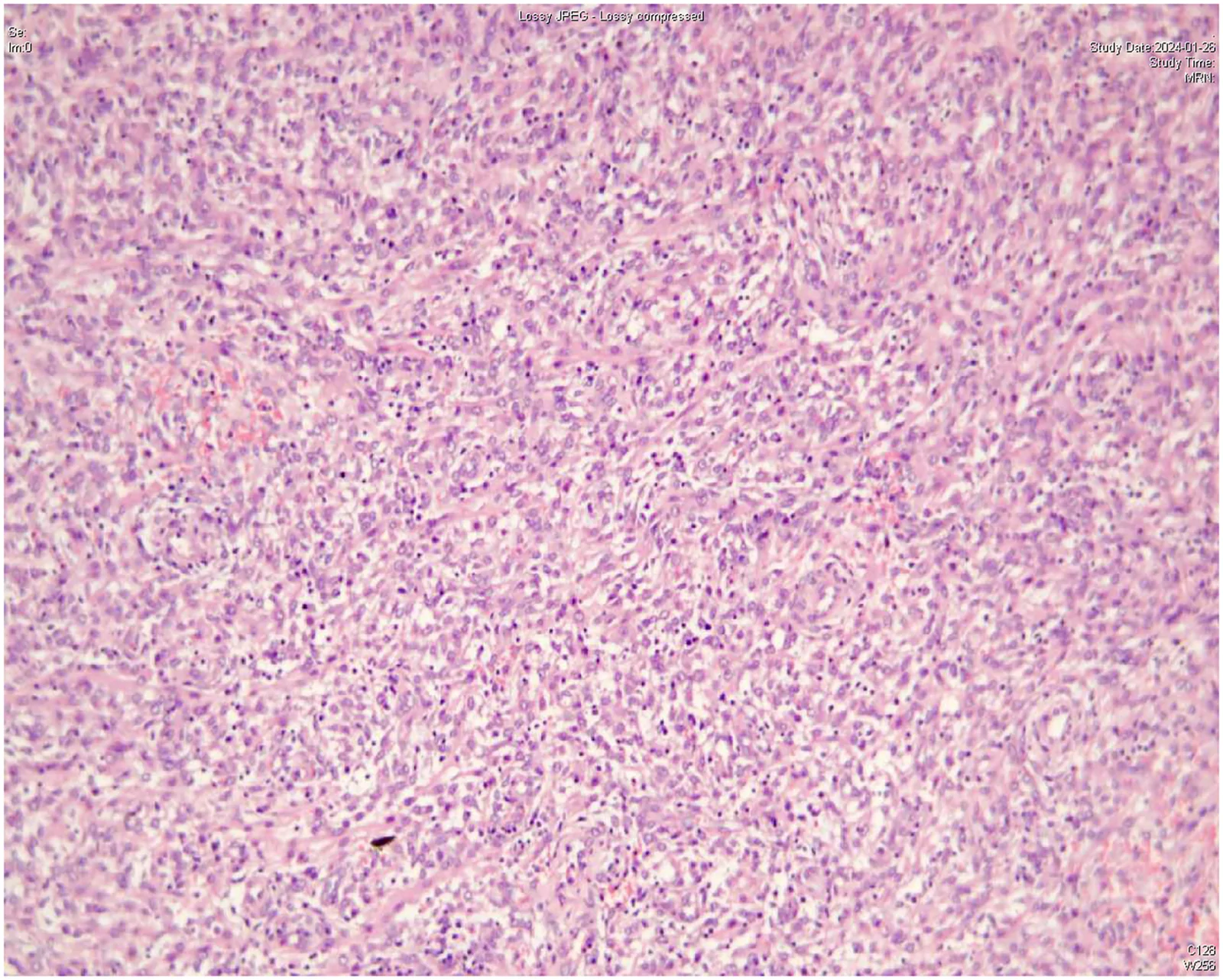
Postoperative histopathological features of the resected prostatic low grade leiomyosarcoma. Hematoxylin eosin staining shows spindle shaped tumor cells.

**Figure 2:**
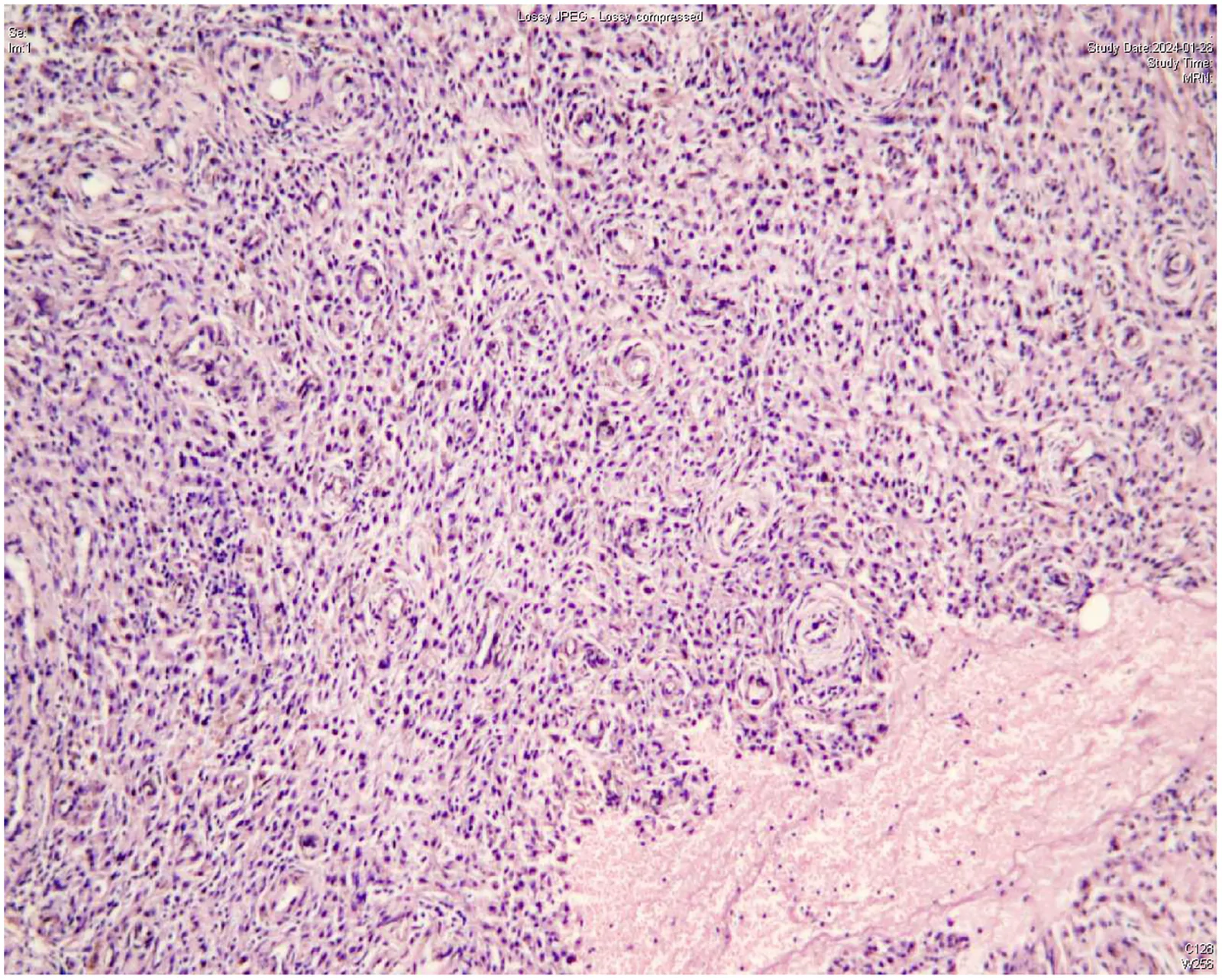
Immunohistochemical staining of low grade leiomyosarcoma. Tumor cells are positive for H caldesmon, SMA and vimentin, and negative for CD34, S 100, HMB 45 and ALK p80. The Ki 67 proliferation index is 5%.

Postoperative histopathological examination of the resected prostatic tissue (**Figure 1**) and immunohistochemical studies (**Figure 2**) confirmed the diagnosis of low-grade leiomyosarcoma of prostatic origin. Immunohistochemically, the tumor was positive for H-caldesmon, SMA, and vimentin; negative for CD34, S-100, HMB-45, and ALK-p80; with a Ki-67 proliferation index of 5%.

**Figure 3:**
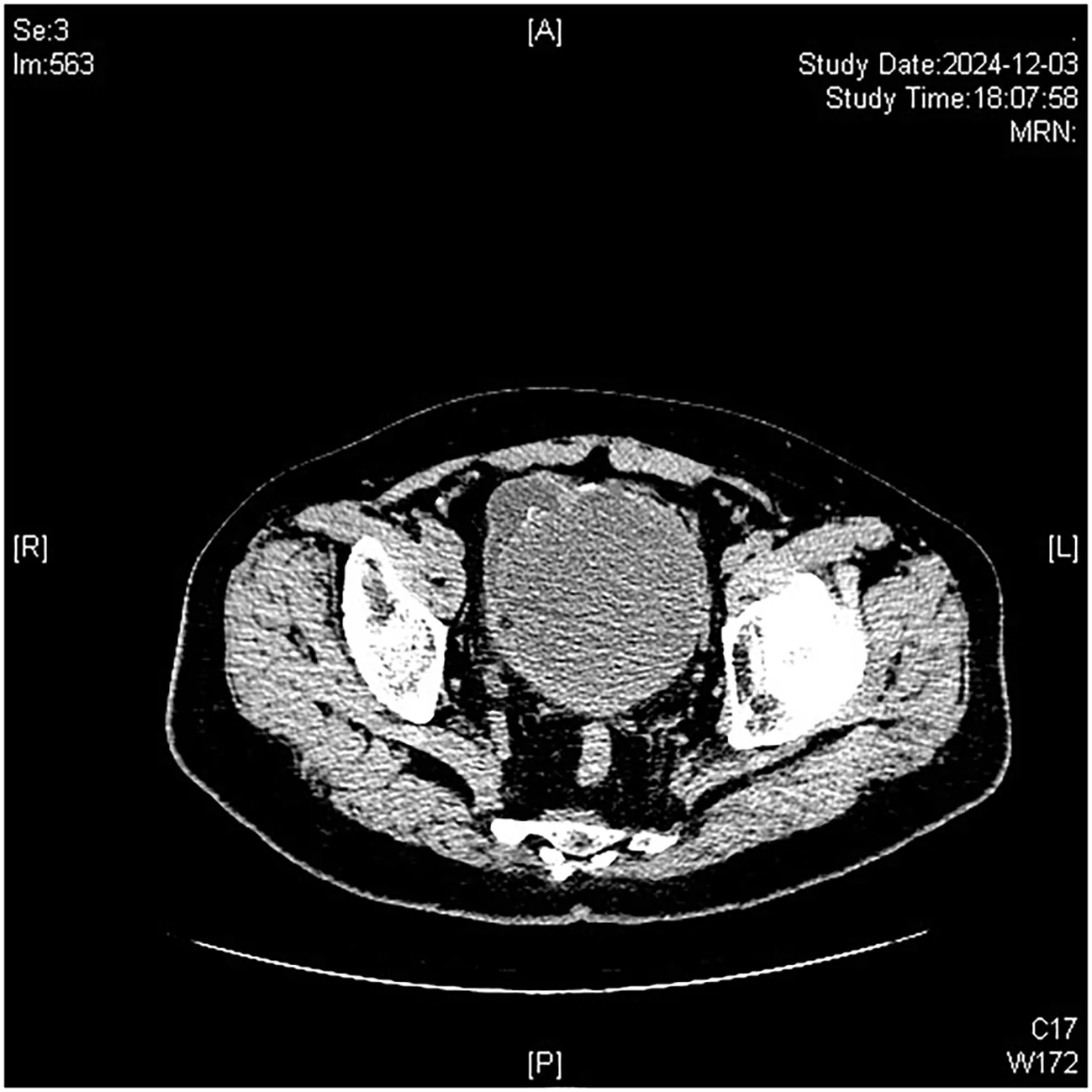
Postoperative abdominal CT scan showing a bladder diverticulum with calcification (arrow) measuring 5.2 × 2.4 cm.

The patient was referred for oncological assessment but declined conventional oncological treatment and subsequently received traditional Chinese medicine. Three months postoperatively, cystoscopy revealed a bladder capacity of approximately 200 mL together with prominent left-lobe prostatic hyperplasia, yet poor visualization secondary to bleeding precluded complete inspection of the bladder. One-year postoperative follow-up CT showed a 5.2 × 2.4 cm right bladder diverticulum with calcification (**Figure 3**) and right hydronephrosis/hydroureter; serum creatinine was 289 µmol/L (normal: 45–105 µmol/L). Following one year of routine catheter exchanges, the catheter was removed after clinical assessment, and the patient reported no recurrence of urinary retention.

## Discussion

Intraperitoneal bladder rupture caused by intravesical gas explosion is an extremely rare complication of transurethral prostate surgery. Although most reported cases are associated with TURP, this event has been scarcely documented after LVP with plasma electrocoagulation (8). This case highlights that the risk may exist even in presumed low-risk procedures, emphasizes the importance of recognizing early warning signs such as gas accumulation at the bladder dome, and provides practical strategies for prevention and management.

The pathogenesis of intravesical gas explosion has been well established in the literature. Tissue pyrolysis generates combustible gases, primarily hydrogen, methane, and carbon monoxide (12). Diathermy produces insufficient oxygen for ignition; instead, atmospheric oxygen (21% oxygen) enters the bladder via the resectoscope sheath, improper use of the Ellik evacuator, or the irrigation system (10, 13). Once atmospheric oxygen is introduced, it mixes with the accumulated hydrogen to form an explosive gaseous mixture (10). The electrical spark from the diathermy loop or coagulation electrode triggers a sudden exothermic reaction (13). Although GreenLight laser differs from monopolar or bipolar resection, subsequent plasma electrocoagulation can generate an electrical spark that may act as an ignition source in a gas-rich environment.

In the present case, scattered gas bubbles were noted at the 12 o’clock prostatic fossa near the bladder neck prior to the explosion. Whether these bubbles represented a transient event or simply entrained air from irrigation or instrument exchange is unclear. No gas-composition analysis was available to confirm a definitive causal link between the bubbles and explosion. Given that plasma resection followed LVP initiation in this case, combustible gas cannot be attributed to either procedure alone or their combined effect.

Harriman et al. documented a case of prostatic capsular perforation with urinary extravasation, presenting as bilateral thigh urinomas and osteitis pubis, following LVP (14). Similarly, Farag et al. reported a case of bladder perforation during LVP that resulted in rhabdomyolysis and acute renal failure (15). These reports, together with our case, illustrate that a spectrum of rare but potentially serious complications may occur following LVP. Unlike some previously reported cases where patients recovered without significant sequelae (8, 12), our patient experienced long-term bladder capacity reduction and persistent dysuria.

Previous reports note a potential link between bladder diverticula and higher bladder-injury risk during transurethral procedures, possibly from focal wall thinning or impaired distensibility (16). Though preoperative ultrasonography showed no bladder diverticulum, postoperative CT identified one. Plausible causes are a pre-existing lesion missed due to limited ultrasound sensitivity, a defect secondary to the explosive injury or surgical repair, or an incidental finding unrelated to the explosion. Without comparative intraoperative imaging and diverticulum-wall histology, its origin and clinical significance remain speculative.

Our recommended preventive strategies comprise evidence-based measures and practical precautions from our institutional experience. Literature-supported recommendations include use of a closed bladder irrigation system (15), moderate electrocautery power settings (8), and prompt evacuation of visible air bubbles (17). By contrast, measures including limiting total operative time to <90 minutes, Trendelenburg positioning, and effective surgeon-anesthesiologist communication stem from our clinical reasoning and institutional practice. In practice, this means that any visible gas bubbles should be actively evacuated before activating the coagulation electrode, particularly when the electrode is positioned within or immediately adjacent to the gas-containing area.

### Limitations

This single-center case report has inherent limitations in generalizability. The source of the flammable gases, whether from LVP or electrocoagulation, remains unclear without intraoperative gas analysis. No intraoperative images were captured due to the emergent nature of the repair. Additionally, preoperative uroflowmetry and bladder capacity data were unavailable, limiting functional assessment.

### Conclusion

Intravesical explosion with full-thickness intraperitoneal bladder rupture is an extremely rare complication that may occur during transurethral prostate surgery involving LVP followed by electrosurgical hemostasis. The observation of intravesical gas bubbles before electrosurgical coagulation may provide an opportunity for prevention. When gas bubbles are identified, they should be evacuated before activation of the electrosurgical electrode. Prompt recognition of bladder rupture and timely surgical repair are essential when this complication occurs.

## Data Availability

The original contributions presented in the study are included in the article/supplementary material. Further inquiries can be directed to the corresponding author.
